# Sociocultural incentives for cancer care implementation

**DOI:** 10.1007/s10585-020-10050-2

**Published:** 2020-07-07

**Authors:** Jörg Haier, Jonathan Sleeman, Jürgen Schäfers

**Affiliations:** 1 Comprehensive Cancer Center, Hannover Medical School, Hannover, Germany; 2 Medical Faculty Mannheim, University of Heidelberg, CBTM, Ludolf-Krehl-Str. 13-17, 68167 Mannheim, Germany; 3 Institut für Toxikologie Und Genetik, Karlsruhe Institute for Technology (KIT), Campus Nord, Postfach 3640, 76021 Karlsruhe, Germany; 4 IGP-Institute for Health Sciences and Public Health, Muenster, Germany

## Introduction

The implementation and modification of cancer care systems in low- and middle-income countries (LMICs) to achieve Universal Health Coverage (UHC) for the entire population is usually accompanied not only by intensive and rapid changes in the delivery processes and system structure, but also by severe impact on human resources and sociocultural aspects of cancer care delivery. At the same time this is caused by and results in complex changes in clinical routines, in collaborative patterns among healthcare providers, professions and disciplines, as well as in the behavior of healthcare workers, patients or other stakeholders, and in the organization of cancer care [1]. Since allocation of qualitatively and quantitatively sufficient human resources to the entire population is a major challenge for cancer care in LIMCs, understanding of sociocultural incentives and their strategic use becomes of high importance [2]. These sociocultural incentives include various types of driving motivations that are not directly related to remuneration of healthcare service, such as free housing, access to professional education, social perception and appreciation, among others. Their importance is especially true for the care of patients with metastatic disease, given their vulnerability and particular clinical needs. Here we consider the impact of sociocultural incentives in this context.

## Sociocultural incentives

Sociocultural incentives aim to guide motivations without directly interfering with financial benefits. Although their effects are indisputable and very well known in other contexts, for example known as generation-Y behavior, they are rarely reflected or scientifically analyzed in healthcare systems, and even less for cancer care. However, individuals are very sensitive to this form of incentivization.

Human resource management is a very important area where sociocultural incentives can have intensive motivational effects in cancer care systems. In LMICs, sociocultural incentives in human resource management are even more important, since in these countries quantitative and qualitative availability (sufficient number of healthcare workers with appropriate qualifications), regional distribution of the workforce (shortages in remote and rural areas) and cross-country migration of qualified staff represent major challenges. This is particularly true for the management of advanced cancer stages, due to the complexity of the treatment options and the clinical specialties that are required. In addition, acceptance of cancer care professionals and modern treatment strategies by the targeted population often depends on sociocultural factors. For example, during palliative cancer care the integration of healthcare workers into social structures, their spiritual acceptance and access to education and knowledge are not related to financial benefits, but have to be considered as key success factors for cancer care implementation.

Successfully motivating and retaining cancer care workers at all professional and non-professional levels is critical for the effective performance of cancer care systems. In many LMICs a shortage of cancer care professionals and low levels of staff motivation in rural and remote areas pose challenges to the provision of equitable cancer care delivery. Frequently this relates to inadequate communication with patients, inappropriate risk selection, avoidance of requisite care provision (e.g. with regard to the number and complexity of treatments) and ineffective employment of resources. Together, these issues result in a significantly impaired quality of clinical care.

Sociocultural incentives are difficult to balance with regards to potential conflicts of interests, which demands intensive and independent monitoring of their effects. Targeted and unintended effects are difficult to differentiate. Public acceptance and an ethical discussion of sociocultural incentives in cancer care would appear to be mandatory for successful implementation. It is also important to note that sociocultural incentives cannot usually replace financial incentives. Nevertheless both types of incentive support and usually even synergistically potentiate each other (Table 1).

**Table 1:** Comparison of financing schemes and sociocultural incentives on cancer care delivery processes and performance

Types of incentives	Covered population	Number of treated cases	Number and complexity of procedures	Risk selection	Accessibility of healthcare structures (regional distribution and underserved patient groups)	Barriers for healthcare usage (inappropriate patient selection)	Clinical quality of care	Efficiency of resource usage
Fixed salaries	Min	Min	Min	Max	Adjust	Max	Min	Min
Capitation	Max	Min	Min	Max	Max	Adjust	Min	Min
Case related payment	Max	Max	Min	Adjust	Adjust	Min	Max	Max
Procedure related payment	Max	Max	Max	Max	Adjust	Min	Min	Max
Sociocultural incentives	Max	Max	Adjust	Adjust	Max	Adjust	Adjust	Max

Min/Max—trend to minimize or maximize; Adjust—effects depend on integrated risk adjustment

Explicit rules for human resource management coupled with transparency in selection and implementation of appropriate incentives should be considered as a central organizational task during health system development in LMICs (at the meso- or macro-levels), in order to encourage physicians and other cancer care professions to work in the public sector, and to improve the availability and accessibility of cancer care delivery. Such transparency in itself may act as an incentive both for cancer care professionals and for the population. As an example, for various groups of cancer care professionals in LMICs, the provision of basic government housing has a great impact on the probability that these professionals will choose a job at a public healthcare facility. Similarly, the provision of formal education opportunities, as well as the availability of equipment and medicine at the healthcare facility also act as important incentives. However, the impact of these measures can vary according to the stage of life of the cancer care professionals, and incentive packages should be tailored accordingly.

Volunteer community healthcare workers (CHWs) are a major resource in LMIC, and their integration into cancer care (such as palliative care for advanced cancer stages) can vary regarding worktime (part time or full-time) and remuneration. Both volunteer and remunerated CHWs have the potential for positive impact in the healthcare system, and can support cancer care availability and accessibility. However, whether this potential is achieved is dependent on various sociocultural incentives. For example, the acceptance of CHWs as important providers of cancer care in LMIC is affected by the stakeholders’ perception of their roles. In addition, predictability of financial rewards for CHWs appears to enhance their performance and their sustainable integration into the delivery processes. Moreover, non-financial incentives, such as community appreciation, anticipated future rewards or professional supervision and training, can support reliance on volunteers’ cancer care participation [3]. For example, well-trained, supervised volunteers, and full-time CHWs who receive regular payment or a combination of training and payment, are more likely to engage in basic cancer care tasks within the community. However, programs that utilize minimal economic incentives for motivating part-time CHWs tend to be limited in focus, with financially incentivized activities playing a central role [4]. If the motivation of volunteer and remunerated CHWs in the cancer care delivery process through sociocultural incentives is neglected, there is a high risk of creating implementation barriers for the provision of a sufficient quality of care, especially in underserved regions.

The migration of cancer care professionals is influenced by macro-, meso-, and micro-level factors that are related to the political, economic, and historic development of LMICs. This migration can occur as professional outmigration from LIMCs with employment in other countries, or as educational mobility from the LIMC with return to the country of origin. Insufficient incentives to remain in or return to LMICs are key reasons for the migration of younger generation professionals in particular. These incentives relate to financial issues, but also to a large extent to sociocultural factors, such as security, licensing-related ability for full practice (acceptance of international degrees) and career opportunities. Stakeholders argue in different manner about educational issues, such as brain drain (negative consequences) or knowledge and technology transfer (positive results). The negative perception is caused by mainly look at concomitant loss of investment into human capital that affects the quality of cancer care delivered, especially in rural areas. On the other side, if young professionals from LIMCs are trained abroad, then return to their home countries, they have the potential to transfer knowledge from the educating host environment to the LIMC. However, the bureaucracy in many LMICs, professional competition, as well as barriers to the acknowledgement of international qualifications frequently impede knowledge transfer, which is counterproductive given the shortage of educated cancer care professionals in LMICs. Additionally, international opportunities have commercialized healthcare education, compromised its quality, and stripped LMICs of skilled learning facilitators, in addition to the many negative social aspects that are associated with the migration. Improvement of suitable socioeconomic incentives for cancer care professionals that requires time and resources is mandatory if this loss of human resources is to be reduced. Importantly, the concomitant massive expansion in education and training due to mobility induces professional growth, creating a paradoxical situation in underfunded healthcare systems. This results on one side in underservice, especially in rural and remote areas, but at the same time in underemployment and outmigration due to lack of resources to guide incentives in more advanced, urban regions.

## Responsibilities for the cancer care community

When considering the importance and complex effects of sociocultural incentives, it becomes clear that the entire cancer care community including all professionals groups, providers, organizations and policy makers needs to focus more on balancing the various types of incentives for all participants in the cancer care processes [5]. Reducing negative sociocultural incentives and barriers as well as setting supportive motivational frameworks can be targeted esp. by political stakeholders without requiring large financial investments in the economically limited environment. Transparency, public discussion and strategic implementation of sociocultural incentives should be considered as a prerequisite for UHC to be achieved, with full coverage cancer care for the entire population in LMICs. If this is ignored or insufficiently considered, vulnerable populations in particular, such as patients with advanced metastatic cancer, will suffer from a lack of available, accessible, acceptable and affordable healthcare. High income countries therefore have a special responsibility to target their development aid and supportive programs towards sustainable human resource management in LMICs. For cancer care in LMICs, the availability of educational activities and educational mobility needs to be guided appropriately to avoid unintended migration of cancer care professionals out of underserved regions.

In conclusion, it is clear that investigating, setting and monitoring sociocultural incentives in cancer care is becoming one of the most challenging tasks for the cancer care community in LMICs. It is vital that this challenge is met if adequate and equitable healthcare is to be provided across entire populations, in particular for vulnerable patient groups such as those with metastatic disease.
